# Evaluation of serum anti-Müllerian hormone levels in women with Hashimoto thyroiditis in the reproductive age

**DOI:** 10.3906/sag-2012-177

**Published:** 2021-04-30

**Authors:** İlknur ÖZTÜRK ÜNSAL, Sema HEPŞEN, Pınar AKHANLI, Murat ÇALAPKULU, Muhammed Erkam SENCAR, Ali YALÇINDAĞ, Erman ÇAKAL

**Affiliations:** 1 Department of Endocrinology and Metabolism, Dışkapı Yıldırım Beyazıt Training and Research Hospital, University of Health Sciences, Ankara Turkey; 2 Department of Biochemistry, Dışkapı Yıldırım Beyazıt Training and Research Hospital, University of Health Sciences, Ankara Turkey

**Keywords:** Hashimoto thyroiditis, anti-Müllerian hormone, ovarian reserve, miscarriage

## Abstract

**Background/aim:**

Autoimmune thyroid disease in women is associated with subfertility and early pregnancy loss, and patients with primary ovarian insufficiency have a high prevalence of thyroid autoimmune disorders. The aim of this study was to investigate the association between Hashimoto thyroiditis (HT) and ovarian reserve.

**Materials and methods:**

Levels of serum thyroid stimulating hormones, thyroid autoantibodies, and anti-Müllerian hormone (AMH) were measured in women with HT and a healthy control group between 2018 and 2019.

**Results:**

Evaluation was made of 108 premenopausal women with HT, and a control group of 172 healthy females with normal antithyroid antibody levels and thyroid function. Serum AMH levels were determined to be significantly lower in the HT group compared to the control group.

**Conclusion:**

Ovarian reserve evaluated by serum AMH concentration is affected by thyroid autoimmunity independently of antithyroid antibodies type or titers.

## 1. Introduction

In women of reproductive age, thyroid dysfunction is the most common endocrine disorder and the most prevalent cause is thyroid autoimmunity [1]. Thyroid peroxidase antibodies (TPOAb) are present in almost 90% of Hashimoto thyroiditis (HT) cases. In reproductive age women, the prevalence of TPOAb is 8%–14% [2]. Antithyroglobuline antibodies (TgAb) are found in 70% of patients with HT. Thyroid autoimmune disease prevalence varies between 5% and 15% in women of reproductive age [3]. The reproductive system is directly affected by thyroid dysfunction as oocytes are affected through thyroid hormone receptors on the surface of these cells [4], and there is an indirect effect through the increased prolactin secretion and disrupted GnRH function [5,6]. It has also been reported that thyroid autoimmune disease may be associated with general autoimmunity, resulting in premature ovarian failure. Nevertheless, there has still not been full clarification of the main pathophysiology linking ovarian reserves with TPOAband thyroid hormones [6,7].

The complex clinical phenomenon of ovarian reserve is influenced by age, genetics, and environmental variables. Biochemical basal and provocative tests and ultrasound imaging of the ovaries are used in ovarian reserve tests. In current clinical practice, the markers most often used are basal follicle stimulating hormone (FSH) levels, antral follicle count, and anti-Müllerian hormone (AMH) [8]. AMH is produced by the granulosa cells of ovarian antral follicles. It indicates both the number of follicles in the follicle pool, and early antral follicles. As age increases, AMH production decreases, so it may be used for evaluation of the ovarian reserve [1,2,9]. As AMH is relatively stable throughout the menstrual cycle in normo-ovulatory women there is no advantage to assessment made by measuring the hormone on a specific day of the menstrual cycle. General guidelines have reported the 5-year age group classifications of the lower limits of age-appropriate serum AMH values as: 0.5 ng/mL at 45 years, 1 ng/mL at 40 years, 1.5 ng/ mL at 35 years, 2.5 ng/mL at 30 years, and 3.0 ng/mL at 25 years [8].

Previous studies have shown that thyroid autoimmune disorders have been detected in 12%–33% of patients with premature ovarian failure [10]. Therefore, infertile patients with autoimmune thyroid diseases may have decreased ovarian reserves. In this case, if thyroid autoimmunity has an effect on follicular growth and development, it may also affect AMH concentrations, independently of female age. The aim of this study was to investigate the correlation between thyroid status, thyroid autoantibodies, and AMH levels in reproductive-aged women.

## 2. Materials and methods

### 2.1. Study population

This retrospective case-control study included a total of 108 patients with euthyroid HT, with regular menstrual cycles ofbetween 25 and 35 days, both those using and not using levothyroxine, and 172 age-matched healthy female volunteers. Patients were excluded if they were aged <18 years or >40 years, had a history of autoimmune disease or polycystic ovary syndrome, or weere taking any adjuvant treatment such as glucocorticoids or anticoagulants. The control group was formed of 172 healthy, euthyroid women with regular ovulatory cycles. The study was conducted in the Endocrinology Department of Dışkapı Yıldırım Beyazıt Training and Research Hospital between January 2018 and December 2019. Demographic data were collected together with clinical details and laboratory values. 

### 2.2. Clinical, biochemical, and hormonal evaluation

Baseline demographic data, clinical characteristics (pregnancy and menstrual cycle history), duration of hypothyroidism, and medications were recorded for all study subjects. Hormonal assays measured in the early follicular phase included serum AMH, free thyroxine (fT4), thyroid stimulating hormone (TSH), thyroid autoantibodies, follicle stimulating hormone (FSH), luteinizing hormone (LH), estradiol (E2), and prolactin (PRL). Blood samples were taken from all patients between 8:00 am and 11:00 am after a 10-hr overnight fast. The serum AMH concentrations were determined fully automated by electrochemiluminescence immunoassay (ECLIA) (Elecsys Cobas e 411-601-602, Roche Diagnostic, Basel, Switzerland, catalog number 0631076 190). AMH levels <1.00 ng/mL were accepted as low. Serum fT4, TSH, TPOAb, and TgAb concentrations, FSH, LH, E2 and PRL levels were measured using ACCEESS 2 immunoassay system (Beckman Coulter, Brea, CA, USA, catalog number 81600N, serial number 509248 and 50992) (reference ranges: fT4, 0.82–1.6 ng/dL; TSH, 0.38–5.33 mIU/L; prolactin, 3.34–26.72 ng/mL). The reference range for TPOAb and TgAb was 0–9and 0–4 IU/mL, respectively at our hospital.

### 2.3. Statistical analysis

Statistical analyses were performed using IBM SPSS Statistics for Windows, version 21.0 software (IBM Corp., Armonk, NY, USA). The variables were investigated using visual (histograms, probability plots) and analytical methods (Kolmogorov–Smirnov/Shapiro–Wilk tests) to assess conformity to normal distribution. The Mann–Whitney U test was performed to compare age, abortus, TSH, fT4, TPOAb and TgAb levels and positivity rates, FSH, LH, estradiol, prolactin, and AMH levels. The Mann–Whitney U test was applied in the comparisons of the AMH levels of the patients classified according to the number of positive thyroid autoantibodies. Descriptive analyses were presented using median values and the interquartile range (IQR) of the 25th and 75th percentiles for nonnormally distributed variables. A p-value <0.05 was accepted as a statistically significant difference. An overall 5% type-1 error level was used to infer statistical significance. While investigating the associations between AMH level and other variables, correlation coefficients and their significance were calculated using the Spearman test. Multivariate logistic regression analysis was performed to determine independent predictors of a low AMH level. The relation between categorical variables were analyzed using chi-square test.

## 3. Results

Evaluation was made of 108 patients with HT with a median age of 32 years (IQR; 27.3–38) and 172 control subjects with a median age of 31 years (IQR; 25–37) (p = 0.209). Of the HT patients, 38.9% (42/108) were using levothyroxine. The median disease duration of the patients was 4 years (IQR; 1.9–6.3). The majority of the study subjects had a history of at least one pregnancy, and miscarriage was reported in 8 (7.4%) HT patients and 4 (2.3%) of the control group (p = 0.041).The median TSH level was 3.1 mIU/L (IQR; 1.5–4.6) in the HT patient group and 2.1 mIU/L (IQR; 1.4–2.9) in the control group. The TSH levels of the patients were found to be significantly higher than those of the control group (p = 0.001). The fT4 levels were similar in both groups (p = 0.497). The median TSH was determined as 2.8 mIU/L (IQR 25–75; 1.56–4.65) in patients with euthyroid HT who were not receiving levothyroxine replacement.

The median TPOAb level of the patient group was 102 (94.4%) IU/mL and TgAb level was 46 (45.1%) IU/mL. The TPOAb and TgAb levels of the patient group were significantly higher than those of the control group (p < 0.001 for each). The FSH, LH, estradiol, and prolactin levels of the patients were similar to those of the control subjects (p = 0.684, p = 0.854, p = 0.064, p = 0.162; respectively). The median AMH level was 1.53 ng/mL (IQR; 0.77–3.6) in the HT group and 2.3 ng/mL (IQR; 1.1–3.8) in the control group. AMH levels were found to be lower in the HT patients compared to the control group (p = 0.047) (Figure). AMH levels in 35.2% (n = 38) of HT patients and in 23.8% (n = 41) of the control group were determined to be <1 ng/mL (p = 0.04). The baseline data, laboratory parameters, and AMH levels are presented in Table 1. 

**Figure:**
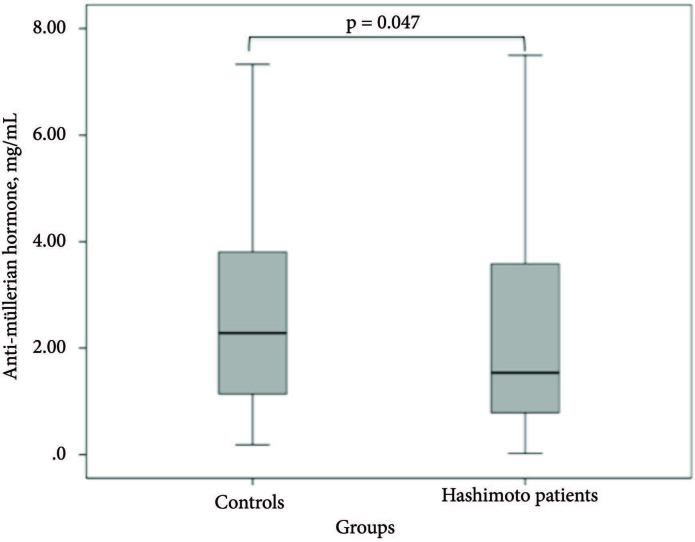
The graphs of anti-Müllerian hormone levels of the groups.

**Table 1:** Baseline data and laboratory parameters belonging to Hashimoto thyroiditis patients and controls.

	Hashimoto patients	Controls	p value
Number,n	108	172	
Age, years	32 (27.3–38)	31 (25–37)	0.209
LT4 usage, n (%)	42 (38.9)	-	-
Disease duration, years	4 (1.9–6.3)	-	-
Patients with miscarriage, n (%)	8 (7.4)	4 (2.3)	0.041
Abortion number, n (%)	1 (1–1)	1 (1–2)	0.642
TSH (mIU/L)	3.1 (1.5–4.6)	2.1 (1.4–2.9)	0.001
fT4(ng/dL)	0.95 (0.83–1.1)	0.93 (0.8–1.1)	0.497
TPOAb(IU/mL)	195 (72–402)	0.7 (0.4–1.1)	<0.001
TPOAb positivity, n (%)	102 (94.4)	0 (0)	-
TgAb (IU/mL)	92.2 (0.9–158)	0.9 (0.9–0.9)	<0.001
TgAb, positivity, n (%)	46 (45.1)	0 (0)	-
FSH (IU/L)	4.3 (6.1–8.1)	6 (4.1–8)	0.684
LH (IU/L)	5.3 (3.2–7.4)	4.8 (3.3–7.3)	0.854
Estradiol (ng/L)	75 (45–106)	54 (36–99.9)	0.064
Prolactin (ng/mL)	11.6 (8.6–14.5)	12 (9–16.6)	0.162
AMH (ng/mL)	1.53 (0.77–3.6)	2.3 (1.1–3.8)	0.047
AMH<1 ng/mL subjects, n (%)	38 (35.2)	41 (23.8)	0.04

Categorical data demonstrated with numbers and percentages (%). Other variables were presented as medians (interquartile ranges 25–75).

The AMH level was found to be negatively correlated with age (r = –0.489, p =< 0.001). There was no relation between AMH level and TSH, fT4, TPOAb and TgAb levels, and disease duration (Table 2). In the multivariate logistic regression analysis, only age (OR: 0.738, 95% CI: 0.595–0.916, p = 0.005) was found to be an independent risk factor for low AMH level in patients with euthyroid HT. No significant difference in AMH levels was determined between subjects who had or had not had a miscarriage and those who were using or not using levothyroxine (p = 0.366, p = 0.734; respectively). There was no difference in AMH levels between patients who had one (either TPO antibody or Tg antibody) or two (both TPO antibody and Tg antibody) positive thyroid autoantibodies (p = 0.543). No significant difference was determined in AMH levels between HT patients who were TPOAb(+) and TgAb(–) vs. TPOAb(–) and TgAb(+) (p = 0.163, p = 0.992; respectively). The number of patients with AMH level <1 ng/mL was similar in the subgroups according to TPOAb or TgAb positivity (p = 0.098, p = 0.744; respectively).

**Table 2:** Correlation analysis results of AMH levels and other parameters.

	R	p value
Age	–0.489	<0.001
Disease duration	–0.055	0.729
TSH level	0.023	0.700
fT4 level	0.016	0.796
TPOAb level	–0.084	0.173
TgAb level	–0.047	0.471

## 4. Discussion

The main finding of this study was lower AMH levels were determined in women with HT than in the age-matched healthy women. No correlations were found between AMH level and TSH, fT4, TPOAb and TgAb levels, and disease duration. Menstrual regularity and ovarian reserve play a critical role in achieving pregnancy in reproductive-age women. Premature ovarian failure (POF) is gonadal failure defined by clinical and laboratory findings before the age of 40. AMH is accepted as a reliable marker for the quantitative evaluation of ovarian reserve, and serum AMH concentration varies with ageing of the female [11]. Ovarian expression of AMH starts in fetal life, reaches a peak at puberty, starts to decline in adulthood and does not occur after menopause. In women with normal menstrual cycles, levels of serum AMH have been shown to decrease before FSH rises [12].

Cellular immunity and autoimmune process abnormalities play a role in the autoimmune etiology of POF. There has been stated to be a personal or family history of autoimmune diseasein 80% of females with idiopathic POF, high titers of thyroid autoantibodies in 50%, and antiovary antibodies in 20%. HT is the most frequently seen disease accompanying POF in adult women [13–16]. The hypothesis of this study was that thyroid autoimmunity may suppress follicle development and would therefore reduce the ovarian reserve in patients with HT. It was assumed that when antithyroid antibodies are present, they may cause antibody-mediated cytotoxicity in the growing ovarian follicle and damage to the maturing oocyte, thereby leading to diminished quality and developmental potential. In the hypothesis of cross-reactivity, it has been suggested that thyroid autoantibodies alter fertility by targeting zonapellucida, human chorionic gonadotropin receptors and other placental antigens [17]. Weghofer et al. reported thyroid autoimmunity at the rate of 11.1% and serum AMH concentration was 1.3 ± 2.0 ng/mL in 225 women who underwent IVF. In that study, the mean AMH levels were determined to be significantly higher in women with TSH <3.0 μIU/mL compared to those with TSH ≥3.0 μIU/mL (p = 0.02) [1]. It was concluded that there was robust statistical evidence that thyroid function in the euthyroid range has a more significant role in the effect on the ovarian reserve than thyroid autoimmunity. However, in the current study, median TSH was 2.8 mIU/L (IQR 25–75;1.56–4.65) in patients with HT who did not use levothyroxine. In the 2012 American Association of Clinical Endocrinologists (AACE) and America Thyroid Association (ATA) cosponsored guidelines, it is stated that the upper limit of a third generation TSH should be considered as 4.12 mIU/L in iodine-sufficient areas. Morover, approximately 20%–26% of the population could be considered hypothyroid if the upper limit of the normal range were decreased to 2.5–3.0 mIU/L [18].

Kurado et al. showed that AMH levels in women with Hashimoto’s thyroiditis were improved with LT4 treatment, but there was no correlation between thyroid antibody titers and serum AMH levels during LT4 supplementation [19]. In contrast in the current study, no differences were determined in serum AMH concentrations of HT patients using or not using levothyroxine (p = 0.734). In a study of euthyroid HT adolescents, Pirgon et al. demonstrated that AMH levels were significantly higher in the HT group than in the control group [17]. In another study, serum AMH levels were determined to be significantly higher in women with HT than in the control group [12]. These results suggested that there could be a common etiological link in HT and polycystic ovary syndrome (PCOS), therefore PCOS patients were not included in the current study.

As expected, there was determined to be a negative correlation between serum AMH levels and age in this study (r= –0.489, p =< 0.001). However, there was no significant correlation of AMH concentration with the levels of TgAb or TPOAb and TSH. Similarly, Chen et al. demonstrated a relationship between positive TPOAb and idiopathic low ovarian reserve in Chinese women, but this was not related to TPOAb levels [20]. Osuka et al. reported that thyroid autoantibodies are not likely to affect ovarian reserve in euthyroid women with a TSH level within the normal range, but elevated TSH levels may be involved in decreasing serum AMH levels [11]. Saglam et al. reported lower AMH levels in euthyroid women with HT compared to an age- and BMI-matched healthy control group (1.16 ± 0.17 vs. 1.28 ± 0.25, p = 0.001 [21]. The results of the current study demonstrated that statistically significantly more HT patients had AMH concentration <1 ng/mL compared to the control group (35.2% vs. 23.8%; p = 0.04). 

Several studies have shown a correlation between subclinical hypothyroidisim and adverse pregnancy outcomes. The results of a metaanalysis showed that the prevalence of miscarriage in patients with subclinical hypothyroidism with autoimmune thyroiditis was significantly higher compared to those with subclinical hypothyroidism only. However, the effect of subclinical hypothyroidism on the risk of miscarriage is unclear. In this study, the rate of miscarriage in patients with HT was statistically significantly higher than in control subjects (p = 0.041), but there was no difference in AMH levels between those with or without a miscarriage (p = 0.366). This suggests that autoimmunity itself may increase the risk of miscarriage. Glinoer et al. reported that thyroid autoantibodies indicate abnormal immune function, which induces miscarriage by unstable placenta implantation [22]. This was explained with 3 hypotheses: 

The first stated that pregnancy loss is not directly linked to the presence of circulating thyroid antibodies, but rather to a more general autoimmune imbalance, which would therefore explain the higher rejection rate of fetal graft. In the second hypothesis, it is assumed that even if there is apparent euthyroidism, the presence of autoimmune thyroid disease could be related tothe decreased thyroid function capacity to adapt sufficiently to the changes of pregnancy with a reduced functional reserve of the thyroid gland. The third hypothesis stated that there was a tendency for pregnancy at an older age in thyroid autoantibody positive women and older women are more prone to pregnancy loss [23]. However, further studies on this matter may be needed to reveal the mechanisms.

There were some limitations to this study, primarily the retrospective design and that antral follicle count could not be evaluated. Most studies of thyroid autoimmunity and ovarian reserve have been conducted on infertile women. This study can be considered to contribute to the limited available literature on ovarian reserve in patients with autoimmune thyroiditis. There have been previous studies in literature examining autoimmune thyroid disease and AMH levels in infertile patients using levothyroxine supplementation. Although the current study also evaluated AMH concentrations in women with euthyroid HT, the patients in this study had not presented because of infertility.

## 5. Conclusion

We observed significant difference for serum AMH levels between healthy women and women suffering from HT. Age is independent risk factor in the decline of serum AMH levels in adult women with autoimmune thyroiditis, and these women must be followed up for premature ovarian failure and infertility. The relationships between autoimmune thyroid disease and reproductive failure have been investigated in numerous studies of infertile women. Nevertheless, due to the difficulties in interpreting the currently available data, there is a clear need for further studies to more comprehensively examine the association between ovarian reserve and HT.

## Informed consent

This study conformed to the Helsinki Declaration. The study was approved by Dışkapı Yıldırım Beyazıt Training and Research Hospital Ethics Committee (No: 10.07.2019–67/08). All participants were informed about the research protocol, and they declared their voluntary attendance by signed written consent. 

## Availability of data and materials

The datasets used and/or analyzed during the current study available from the corresponding author on reasonable request. 

## Contribution of authors

I.O.U, S.H, P.A, M.C, M.E.S participated in data collection, I.O.U, S.H contributed to interpretation of results, data analyzes, A.Y. analyzed biochemical, and hormonal evaluation I.O.U wrote and edited the manuscript, I.O.U, M.C, S.H contributed to the discussion. I.O.U, E.C contributed to study design, reviewed and edited the manuscript. All authors read and approved the final manuscript.
